# The Road to the Beijing Winter Olympics and Beyond: Opinions and Perspectives on Physiology and Innovation in Winter Sport

**DOI:** 10.1007/s42978-021-00133-1

**Published:** 2021-09-27

**Authors:** Jun Wang, Hongwei Guan, Morten Hostrup, David S. Rowlands, José González-Alonso, Jørgen Jensen

**Affiliations:** 1 Department of Exercise Physiology, Beijing Sport University, Beijing, China; 2 Department of Health Promotion and Physical Education, School of Health Sciences and Human Performance, Ithaca College, Ithaca, NY 14850 USA; 3 Section of Integrative Physiology, Department of Nutrition, Exercise and Sports, University of Copenhagen, Copenhagen, Denmark; 4 School of Sport, Exercise, and Nutrition, College of Health, Massey University, Auckland, New Zealand; 5 Centre for Human Performance, Exercise and Rehabilitation, Brunel University London, Uxbridge, UK; 6 Department of Physical Performance, Norwegian School of Sport Sciences, Ullevål Stadion, P.O.Box 4012, 0806 Oslo, Norway

**Keywords:** Exercise physiology, Endurance training, Maximal oxygen uptake, Temperature, Protein, Asthma, Cross-country skiing, Ski patrol

## Abstract

Beijing will host the 2022 Winter Olympics, and China strengthens research on various aspects to allow their athletes to compete successfully in winter sport. Simultaneously, Government-directed initiatives aim to increase public participation in recreational winter sport. These parallel developments allow research to advance knowledge and understanding of the physiological determinants of performance and health related to winter sport. Winter sport athletes often conduct a substantial amount of training with high volumes of low-to-moderate exercise intensity and lower volumes of high-intensity work. Moreover, much of the training occur at low ambient temperatures and winter sport athletes have high risk of developing asthma or asthma-related conditions, such as exercise-induced bronchoconstriction. The high training volumes require optimal nutrition with increased energy and dietary protein requirement to stimulate muscle protein synthesis response in the post-exercise period. Whether higher protein intake is required in the cold should be investigated. Cross-country skiing is performed mostly in Northern hemisphere with a strong cultural heritage and sporting tradition. It is expected that innovative initiatives on recruitment and training during the next few years will target to enhance performance of Chinese athletes in classical endurance-based winter sport. The innovation potential coupled with resourcing and population may be substantial with the potential for China to become a significant winter sport nation. This paper discusses the physiological aspects of endurance training and performance in winter sport highlighting areas where innovation may advance in athletic performance in cold environments. In addition, to ensure sustainable development of snow sport, a quality ski patrol and rescue system is recommended for the safety of increasing mass participation.

## Introduction

**Fig. 1:**
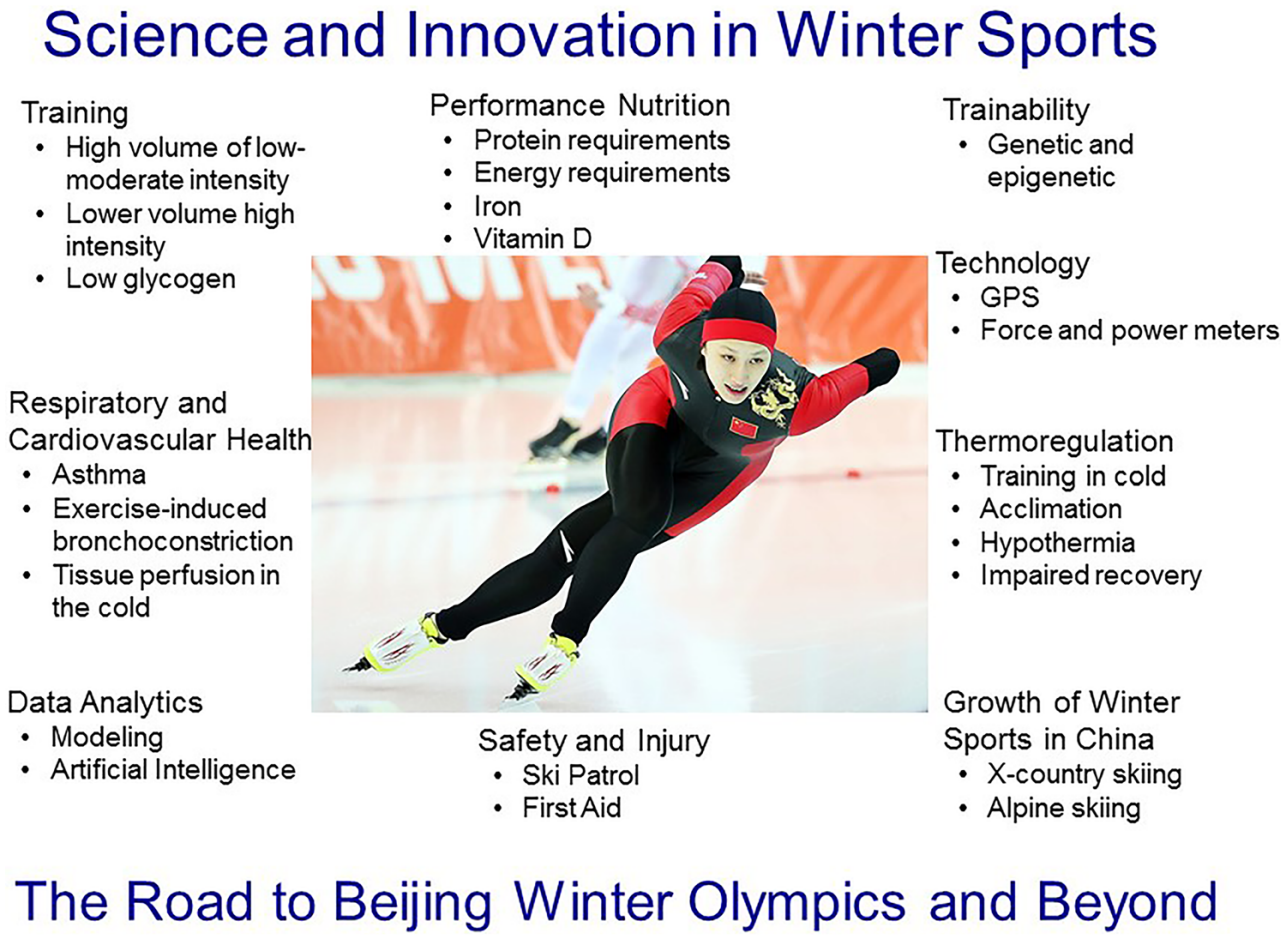
Physiological and technological aspects of performance in winter sport

Beijing hosts the 2022 Winter Olympics and the preparation includes strengthening of research related to winter sport. In winter sport, cross-country skiing represents endurance competitions in its most authentic form, and the sport is very popular in a limited number of countries in the Northern hemisphere, and the athletes perform large volume of training. Cross-country skiing is often performed at low ambient temperatures and much of the training is performed at low-to-moderate intensity with few training hours at high intensity [65, 70, 86]. Body core temperature is generally maintained above 37 °C during training and competition in winter sport, but skin and regional limb tissue temperatures are much lower. The ambient temperatures in the Xinhua Nordic Centre during the Beijing Winter Olympics are expected to be in the low teens (− 12 to − 6 °C). Thus, local tissue temperature could be sub-optimal for normal musculoskeletal function since extreme cold (− 15 °C) has shown to impair cross-country skiing performance [94, 95]. The high weekly training load at low ambient temperatures, frequently coupled with low relative humidity, may also be the reason why such a large proportion of cross-country skiers suffer from asthma or experience asthma-related symptoms, such as exercise-induced bronchoconstriction, hence needing medical treatment with inhaled bronchodilators and glucocorticoids [3]]. High volume of endurance training requires high energy and protein intake; importantly, endurance athletes need ~ 2 g protein/kg per day to maintain nitrogen balance over time [26, 80]. High intake of protein is necessary to stimulate recovery and may benefit muscle adaptation and endurance performance [5]. The training for winter sport athletes needs to take these physiological aspects into consideration to reach elite level (Fig. 1 for overview).

The 2022 Winter Olympics in Beijing provides the impetus to promote winter sport and increase recreational participation—particularly in China. As the hosting nation, China has made several initiatives to promote winter sport. While the ice-skating disciplines holds high international standard, including Olympic medallists, new adult athletes are recruited into cross-country skiing. Most likely, new training strategies may be required to optimise performance in athletes converting to cross-country skiing from other types of sport. Introducing skiing for mass participation in China will require considerable development of infrastructure, including facilities and rescue organisations, but also allow China becoming a significant winter sport nation. Many reviews have addressed on narrow topics related to performance in winter sport [10, 11]. However, there is a gap of knowledge integrating physiological aspects of winter sport in holistic perspectives. This paper addresses physiological topics related to endurance training and performance in winter sport in a broader perspective. The aim of the paper is to discuss the physiological aspects of endurance training and performance in winter sport highlighting areas where innovation may advance athletic performance in cold environments. Finally, requirements to infrastructure allowing China to increase recreational winter sport is addressed briefly.

## Strategies for Endurance Training

Training for endurance competitions aims to enhance an athletes’ ability to sustain prolonged, intense exercise. Training principles in endurance sport have changed over the years, and modifications will continue—although revolutions are not expected. Endurance training can be separated into continuous or interval-based exercise, in which both forms can be performed at various intensities, durations, and sequences. Hence, numerous training programs can be composed, as illustrated by the huge variation between athletes of different sport, age, sex and stage in career.

A hundred years ago, Paavo Nurmi (1897–1973, Finland) included much continuous training at low intensity and even walking in his preparation [22]. The training was successful for Nurmi and his merits include 9 Olympic gold medals and 22 World records. Emil Zatopek (1922–2000; Czech Republic) won the 5000 m, 10,000 m and marathon at the Olympics in Helsinki 1952, and became an advocate for interval running in the late 1940s. Zatopek is famous for running 400 m intervals at high speeds. The 20 × 400 m (interspersed by 200 m at slow speeds in between intervals) corresponds to a volume of 12 km during a commonly performed training session, but he was running up to 60 × 400 m daily in his preparations [36]. Zatopek also performed 10 × 200 m intervals at maximal speed, wherein each 200-m run took a little less than 30 s [36]. Such training can be considered the forerunner for the popular high-intensity interval training (HIIT) [66]. The Norwegian orienteering runner, Egil Johansen (1954-; Norway), was the 1976 and 1978 world champion. Johansen carefully registered his training, which reached 24 h and 260 km weekly during the periods with the highest training load (Unpublished). Most of his training hours were spent at moderate intensity with only a few weekly training hours spent at high intensity. The Scandinavian tradition with high amount of endurance training at low-to-moderate intensity remains common among cross-country skiers [64, 65, 70, 86]. In long-track speed skating, performance times in the 5000 m and 10,000 m have substantially improved over the last 50 years. Improvements have been explained by technological improvements, such as, indoor ovals, klapskates, aerodynamic suits, improved ice preparation, improving economy and athletic performance [51]. Another innovation in endurance training is a shift towards more polarised training [77]; that is, training high volumes in blood lactate concentration zone 1 (low intensity; < 2 mmol) and lower volume of high-intensity (10%–15% in zone 2 and 3; 2–4 and > 4 mmol, respectively) [53].

Cross-country skiers are characterised by high values for maximal oxygen uptake (VO_2max_). Among Norwegian World Champions, the average VO_2max_ values are 84.3 ± 5.2 mL/kg/min for males and 72.6 ± 5.1 mL/kg/min for females [65, 70, 85]. The variation in VO_2max_ is large and exceeding 90 mL/kg/min for some of the best male skiers. Some of the best cross-country skiers train more than 1000 h yearly and 800–900 h are common [69, 85]. Obviously, the majority of such a high training volume needs to be of lower to moderate intensity.

Training studies, however, mostly include non-elite untrained or moderately trained subjects, who perform a period of training with lower volumes than those performed by athletes. One must therefore be cautious when extrapolating findings from such studies to athletes. The fact that HIIT increases VO_2max_ was described 30 years ago [41]. Various forms of sprint interval training and speed endurance training are now common [28] and high intensity training is effective. For example, three weekly sessions of 30-s sprint running for 10 weeks have increased VO_2max_ similar to 60 min running at 75% VO_2max_ [66], however, it is important to realise that short-term training studies (2–12 weeks) may have limited importance for composition of training programs for elite athletes, because training volume is several fold higher in elite endurance athletes. The effect and adaptation to yearlong endurance training should be addressed scientifically in prospective studies in the future (Discussed more thoroughly later).

Cross-country skiers train and compete systematically for many years and reach high VO_2max_ before winning Olympic medals [85]. Although much training is performed at low temperature, there are no training studies where the role of environmental temperature during training sessions have been studied systematically, to the authors' knowledge. Moreover, cross-country skiers perform much training in double-poling, where the major load is on the arms, whereas other work is performed to condition the legs. Therefore, cross-country skiers train a larger muscle mass than runners and cyclists and another question that needs to be addressed scientifically is: does the combination of training sessions focussing on legs (running) and arms (double-poling) allow larger overall training volume and ultimately higher VO_2max_?

Skeletal muscle adaptations to exercise training occur at multiple levels including key cellular and molecular signalling pathways. The AMP-activated protein kinase (AMPK) is for instance thought to be a key signal involved in regulation of mitochondrial biogenesis [24]. Understanding that AMPK is involved in regulating mitochondrial biogenesis allows us to investigate how exercise regulates AMPK. Interestingly, AMPK is activated much stronger by exercise when muscle glycogen content is low [37]. In the 1990s cross-country skiers often drank much carbohydrate during their training sessions allowing high intensity and duration. We now know that adaptation is influenced by carbohydrate ingestion during the training [52]. Today, training with low glycogen is practiced by some athletes either by training two sessions in a day with low carbohydrate intake between sessions, or by training in the fasted state, or by reducing food intake immediately after training. The outcome of such strategies is not clear, but athletes are normally adapting training to improve performance. The diet and training interaction has been documented [14], but future studies should address this topic in particular with timing of recovery nutrition.

The data from training studies in a variety of human populations show large heterogeneity in the increase in VO_2max_ [39] and much research has been done to identify the underlying factors. Although genetic polymorphisms were claimed to explain part of the variance in VO_2max_ in the HERITAGE study [7], to date no genes have been proven to predict VO_2max_. Interestingly, three Norwegian brothers currently compete successfully in 1500–5000 m. Although children inherit their mother's mitochondrial DNA, two brothers can carry different alleles from both parents and the similarities in nuclear DNA will not be profound. A genetic component of endurance capacity is clearly illustrated with selective breeding in rats, wherein rats with high running capacity or other traits can be developed [34].

These results suggest heredity of endurance capacity, but no genes have so far been validated to predict high aerobic capacity or trainability. Indeed, the heredity of endurance capacity may be epigenetic. However, the fact that endurance training can increase VO_2max_ by 50% in some human populations (particularly individuals with low aerobic fitness) makes it difficult to determine genetic and epigenetic components of endurance capacity and large well-characterised cohort will be required. Therefore, it seems reasonable that elite athletes have genetic predisposition, but high volumes and yearlong training is required to reach top level in endurance sport. Currently, DNA analyses have become much easier and huge amount of data are collected with various technologies (GWAS, exon analyses and complete genome sequencing). No doubt, such data will determine genes that are important for endurance capacity, which again may be useful for the development of training strategies.

## Physiological Limitations to Maximal Oxygen Uptake and Endurance Performance

The factors limiting aerobic power in winter sport athletes are not well defined—in particular when different types of sport and modalities of exercise are considered. Potential limiting factors are ventilation, blood volume, cardiac output, locomotor muscle blood flow, oxygen transport, oxidative capacity in skeletal muscle, and substrate availability [4, 40]. Locomotor limb blood flow and cardiac output are considered important limiting factors for maximal oxygen uptake in normoxic conditions because of their potential negative impact on peripheral tissue and organ oxygen supply [4, 45, 58, 92]. Studies indeed support that the transport of oxygen to active muscles limits oxygen transport capacity and impairs endurance performance in trained athletes [18, 21, 47, 47, 93], but the precise interplay between peripheral and central mechanisms restricting active muscle and systemic blood flow is still a matter of debate [4, 32, 33, 50]. However, elite endurance athletes reach oxygen extraction of 90%–95% and increased oxygen delivery seems necessary to increase VO_2max_ [71].

During strenuous exercise, arterial desaturation of oxygen can occur in athletes with maximal oxygen uptake above 75 mL/kg/min [10, 12], as also observed in cross-country skiers. In prolonged endurance competitions, exercise intensity is traditionally considered to occur below VO_2max_ where exercise-induced arterial oxygen desaturation does not develop. However, competitions in cross-county skiing normally occur in hilly terrain and workload has been estimated to exceed 150% of VO_2max_ in the steepest parts, and the resulting oxygen deficits recovers in the downhills [17]. Therefore, cross-country skiers may experience arterial desaturation in some sections of races. Furthermore, the competitions in cross-country skiing at the 2022 Beijing Winter Olympic Games occur 1635–1686 m above sea level, and such altitude accelerates exercise-induced hypoxemia and the lungs may restrict maximal exercise capacity. That said, it is reasonable to believe that the limiting factors for VO_2max_ and endurance capacity may vary among athletes, and thus knowledge of the individual limiting factors for aerobic and endurance exercise capacity would be useful to optimise individual performance and training loads. Moreover, the capacity to repeatedly work at loads above VO_2max_ and recover fast seems important in modern cross-country competitions. Innovative research into training in cross-country skiing may include testing the effect of training with variable intensities, where periods of very high intensity exercise with substantial anaerobic contribution are interspersed by periods of relative lower intensity allowing oxygen deficit to recover.

## Extreme Environments and Exercise Performance

Winter sport athletes train and compete in extreme ambient conditions, such as severe cold and uncontrolled humidity in outdoor facilities [6, 9, 27]. The Beijing Winter Olympics will take place in February 2022, and the temperatures in the Xinhua Nordic Centre are expected to be in the range of − 12 to − 6 °C. However, the effective temperature could be much lower if wind speed is high due to the wind chill factor. The question of what limits athletic performance in extreme environments is highly complex because of the multiple molecular, cellular, tissue and organ systems involved in the processes underpinning fatigue. That said, different schools of thoughts highlight the significance of the functional alterations occurring in one and/or all the three major organ systems at work during exhaustive exercise; namely, the heart, the brain, and the active skeletal muscles and the role that temperature might play in the fatigue processes [87, 88]. The responses of these organ systems during submaximal and maximal aerobic exercise in extreme cold and extreme heat environments are different, but some common features are apparent. These environments induce large changes in body core temperature (leading to hypothermia or hyperthermia, respectively) and functional alterations in multiple other physiological systems. There is strong evidence that severe hyperthermia and severe hypothermia limit physiological function and exercise performance in extreme environments [6, 9, 68, 94, 95]. This is supported by evidence in isolated skeletal muscle fibres showing significant reductions in maximal tetanic force when muscle temperature either decreases below 33 °C [57] or increases above 43 °C [56] and work in intact humans revealing that leg cooling induces marked decreases in peak power during maximal exercise lasting 20 s, whereas leg heating improves sprinting performance [67]. Whole body hyperthermia, however, impairs the repeated 30 s Wingate tests [13]. The study by Thompson and Hayward [81] is particularly relevant for winter sport, as it conclusively demonstrates that conditions such as wet-cold exposure that leads to hypothermia (core temperature of ≤ 35 °C) drastically impairs exercise capacity.

The consequences of diminished oxygen supply for aerobic metabolism in athletes have been characterised in the exercising muscles and human brain during strenuous exercise under mildly cold and heat stress conditions [18, 21, 87, 89, 90]. Evidence indicates that the heart, the brain, and the locomotor muscles experience some level of restriction in blood flow and oxygen supply during maximal aerobic exercise, but its consequences are greater in active skeletal muscles than in the brain and the heart due to the contracting skeletal muscles’ smaller functional oxygen extraction reserve when approaching exhaustion [19, 20, 87, 89, 91]]. Analogous physiological data under conditions producing hypothermia are currently lacking. However, available evidence strongly supports that both hypothermia and hyperthermia expedite fatigue processes and thus interventions that prevent drastic changes in body core temperature should be considered to optimise physical performance in extreme environments. The impact of thermal clothing on physiological function and exercise performance is an area for further research and innovation.

## Respiratory Complications and Treatment of Winter Sport Athletes

The extreme environmental conditions and the substantial number of weekly training hours with high volumes of respiratory airflow are risk factors associated with the respiratory complications commonly reported by winter sport athletes [79]. Asthma and asthma-related conditions, such as exercise-induced bronchoconstriction are highly prevalent among winter sport athletes and treatment can be challenging [78]. Asthma and exercise-induced bronchoconstriction are characterised by respiratory symptoms, including airway hyper-responsiveness to stimulants, narrowing of the bronchioles, coughing, wheezing, mucus secretion, and shortness of breath. If left untreated, the airway hyper-responsiveness may compromise exercise performance by causing limited airflow as well as increased respiratory muscle work and rating of perceived exertion [55].

The diagnosis of asthma and asthma-related conditions should not only be based on spirometry but more so on various bronchial challenges, as well as blood immunology and atopic testing to characterise a potential asthma phenotype [3]. Athletes with confirmed asthma or an asthma-related condition should be treated in accordance with the GINA guidelines (Global Initiative for Asthma), which is divided into 5 steps based on the disease severity. First-line treatment is inhaled glucocorticoid and β2-agonist as needed. Importantly, the athlete and team doctor should be aware of the anti-doping regulations for substances used in asthma treatment, and a therapeutic use exemption (TUE) may be required for certain anti-asthmatic compounds.

If an athlete complains of respiratory complications but with no apparent objective indication of asthma or asthma-related conditions, the team doctor should consider differential diagnoses, such as dysfunctional breathing or exercise-induced laryngeal obstruction [49]. The latter condition is a prevalent differential diagnosis among athletes and is characterised by supraglottic or vocal cord dysfunction causing a narrowing of the tracheal inlet, hence compromising airflow. Thus, coherent respiratory testing of winter sport athletes should not be neglected. Several innovative steps may help prevent and manage asthma and asthma-related conditions in winter sport athletes, such as face masks to heat and moisture inspired cold air, pre-exercise inhalation of saline solutions to reduce the release of cytokine mediators in the bronchii, and indoor training to control ambient conditions. It still needs to be investigated whether certain types of training (e.g. high intensity interval training) under some extreme environmental conditions are risk factors for the development of respiratory complications.

## Nutrition, Recovery and Muscular Adaptation

Endurance training and competition in elite athletes frequently involves consecutive days with high physiological stress and limited time for recovery. Indeed, the pinnacle elite winter sport endurance athletes—cross-country skiers—train at both high volumes of typically 800–900 h per annum, which include up to 10%–20% at near or above race-pace [69, 70]. Accordingly, inferring diet and supplemental feeding strategies from studies in cyclists and runners who also train in similar ways may assist in dietary interventions to aid in training and subsequent endurance performance. The role of muscle glycogen and dietary carbohydrate in endurance performance was well established by the 1980s [25]. More recent research in the last 20 years has assisted in defining the role dietary protein plays in the molecular processes underlying muscle recovery and training adaptation. Protein provides the amino acids required to replace oxidative losses during exercise [38], to stimulate protein synthesis [62] and modify exercise-mediated gene expression to provide the molecular signal and materials for the tissue repair and remodelling during recovery [61], which includes an overrepresented inflammatory-promyogenic-metabolic response.

Protein requirements—largely based on nitrogen balance (NBAL) [60, 80, 83] and indicator amino acid oxidation (IAAO) methodology [96]—are elevated in male athletes with mean estimates of 1.6–2.0 g/kg body weight per day and in females 1.3–1.7 g/kg body weight per day [8, 60, 80, 82, 96]. Under heavy training or competition conditions (e.g. Tour de France) [8], however, individuals or average athlete cohorts under negative energy balance conditions (e.g. for body composition objectives), may benefit from up to 2.0–3.0 g/kg body weight per day. The co-ingestion of carbohydrate and protein in the form of supplements or targeted meals after endurance exercise generally stimulates a greater rate of glycogen synthesis [97], which offsets increased protein metabolic requirements, at least up to the ingestion of comparatively saturating levels of carbohydrate [31]. After resistance and endurance exercise, between 20 and 40 g of ingested leucine-rich protein (e.g. dairy, soy, egg) maximally stimulates protein synthesis rates [46, 62]; an effect that can be sustained by pulsatile feeding every 2–4 h [2]. However, the response of muscle protein synthesis to ingested-protein dose after endurance exercise is less well studied, with the only study suggesting increased muscle protein synthesis may occur after at least 70 g of protein with added leucine (15–18 g) [62].

Protein ingestion before and after exercise improves subsequent performance, most of the time. However, favourable effects appear to be mostly coupled to relatively positive NBAL associated with the higher protein diet or supplement, relative to a control condition [11, 59, 60, 63, 72, 83, 96]. By contrast, performance is often not clearly affected when the total background diet contains enough protein and energy to meet NBAL and energy balance, respectively [48]. Limited data in female athletes suggest that the average response of recovery performance to supplemental protein is more variable and unclear [1, 59]; however, much more research is required in women to understand the dietary and endocrine parameters underlying the variabilities. There are also no data that we are aware of in winter sport athletes, who may respond somewhat differently associated with exercising in the cold environments, which may affect the response of recovery protein synthesis and breakdown to protein feeding [15, 42], and with low background vitamin D exposure [16, 84], which has been associated with muscle function and may impact upon adaptation to training.

Dietary strategies targeted at enhancing protein metabolism and/or whole-body anabolism may be optimal to support a high training quality and adaptation under periods of energy and protein stress in men undertaking endurance training. Moreover, food derived and/or supplementation of the normal protein dietary intake may support intense endurance exercise performance during high-frequency daily training or competition, typical of an elite endurance athlete. As elite winter sport endurance athletes typically train at durations and intensities similar to cyclists and runners, it is probable that the conclusions drawn will also apply to their athletes, with the caveats that elite athletes are normally less sensitive to intervention efficacy than the typical sub-elite study cohorts, and little is known about winter training conditions (cold, restricted sunlight) and dietary protein related issues around daily protein balance and impact of timed supplemental protein on muscle recovery and performance. These questions are good topics for research and provide potential opportunities for innovation and small worthwhile benefits.

## Translation of Sport Science to Endurance Training in Winter Sport

Physiologists have provided detailed knowledge of how the human body works during acute and chronic exercise to explain overtime improvement in athletic performance and training. The trainers responsible for elite athletes are seldom scientists, and training is normally adapted weekly to optimise load in relation to physical condition. Many countries and professional sport teams have experts in exercise physiology, biomechanics, data analyses, psychology and physical therapy to support the trainers. However, generally experts help the trainers to translate new knowledge from physiology and technology into the training or development of equipment. In some cases, scientists can be involved with elite athletes and for example testing the role of caffeine on performance in elite cross-country skiers [74].

An important innovative aspect in sport science would be conduction of yearlong training studies on elite athletes with large increase in performance. Importantly, the biggest football and cycle teams have physiologist employed and well-equipped laboratories with the ability to conduct such studies to optimise development of performancē. Such research is normally conducted to optimise performance to achieve competitive advantages, but such data are still valuable with some years delay [30]. However, it must be expected that research conducted to optimise performance to achieve competitive advantages will be withheld until no further competitive advantage is apparent. Beijing Sport University has good opportunities to conduct such research since several scientists are connected to the National Teams and are involved in testing. Interestingly, it was recently shown that low energy availability was a serious risk factor for injury in aesthetic gymnastic athletes [43].

An area of research in elite winter sport is the careful and systematic registration of intensity, volume and type of training in elite athletes. New technologies allow precise collection of huge amount of data from training and other activities in elite cross-country skiers and other endurance athletes. For example, registration of heart rate and heart rate variability in relation to training load and power output, Global Positioning System (GPS) tools, and performance tests can be analysed using advanced data analytics and statistical models including artificial intelligence (AI) to obtain in depth understanding of the relationship between training load and physiological responses. Advanced analyses of such big data have potential innovate and optimise training.

Blood samples are sometimes used to monitor physical condition of elite athletes, and in particular when results are not as expected. For example, blood ferritin and haemoglobin concentration are routinely evaluated to test for clinical iron stores or anaemia, which is associated with impaired aerobic endurance performance. Similarly, seasonal blood vitamin D status tracing may be worthwhile in winter sport athletes, as there is some provisional associational evidence with muscle performance. The ratio between testosterone and cortisol has been used for many years, but acute sessions of strength and endurance training substantial increase both hormones [29], and the usefulness of the testosterone/cortisol ratio to assess training is not convincing. Currently, new technologies allow more comprehensive analysis of metabolites (metabolomics) in blood, and the physiological roles of large number of plasma peptides are under investigation. Some of these seem to have important physiological roles and may become useful to prevent overtraining. Recently, the plasma peptide GDF15 has gained much attention and seems important in metabolic regulation [54]. Therefore, physiological research may describe new markers for training, which could be useful to optimise training and avoid overreaching.

Training in the cold may increase glycogen utilisation rate [75]. We are unaware whether this effect is attenuated by chronic adaptation to cold-climate training. Research is also required to check if the metabolic effects of cold-climate training impact acute and chronic protein metabolism. Event fuelling is challenging for cross-country skiers, ski jumpers and skaters may require other dietary interventions to optimise body composition, which is likely to necessitate professional attention to ensuring protein, carbohydrate, fluid, and micronutrient requirements are met during the intervention period [44]. Furthermore, more genetic or epigenetic markers of endurance and trainability will probably be identified in the future. See Table 1 for overview.

**Table 1:** Perspectives on future research in winter sports—in search of marginal gains

Observation or association	Future research perspective
X-country and endurance skating training distribution towards more volume of low-intensity training associated with improved performance	What mechanisms govern performance advantage of increased intensity polarisation?
The best endurance skiers have very high VO_2max_, but the genetic determinants are unknown	Discovery of genes or epigenetic regulators associated with high VO_2max_ or endurance trainability may assist understanding predisposition to top performance
Severe cold can impair performance, but clothing can attenuate cold	The impact of advanced technical thermal clothing on physiological function and performance is an area for research innovation
Asthma and bronchoconstriction are highly prevalent among winter sport athletes training in cold, dry air conditions	Research the use of face masks to heat and moisture inspired cold air, pre-exercise inhalation of saline solutions to reduce cytokine mediators, and indoor training in thermoneutral conditions
Cold muscles syntheses new muscle protein slower, while the effect of cold-climate training on daily protein requirements are unknown	Effects of cold exposure on muscle adaptation and performance, and if cold acclimation to can offset impairment of protein synthesis
Low UVB exposure and vitamin D status may impact on training adaptation and performance	Vitamin D status profiling and impact of supplementation
Technology allows for relatively precise tracking of training stress parameters	Advanced statistical models including artificial intelligence may be useful in identify key training components predicting performance
Traumatic injuries are common in winter sports, yet China has not formal ski safety program	The effects of implementation of a formalised ski patrol/ice safety strategy and system on injury risk and rescue statistics

The table provides an overview perspective of identified research questions and innovations that maybe investigated with particular reference to winter sport performance development and community health

## Winter Sport in China in Years to Come

The 2022 Winter Olympics in Beijing will promote winter sport to the Chinese people, but successful snow sport development includes managing safety issues and the high rates of trauma injuries associated with snow and ice sport [73]. Comparative research on ski participation and injury between the US and China [23], shows the critical importance of establishing a quality ski patrol and rescue system, as well as an education program in China.

As Beijing prepares to host the 2022 Olympic and Paralympic Winter Games, more Chinese participate in winter sport. China has an ambitious goal of encouraging 300 million people to participate in winter sport. Such a goal boosts the winter sport industry, and also increases physical activity and participation rates with benefits to health and fitness. According to the 2020 China Ski Industry White Book [97], the number of ski resorts in China has grown from 50 in 2000 to 778 in 2020 (63 temporarily closed because of COVID-19); individual skier visits also increased from 0.3 million in 2000 to 20.8 Million in 2020. With the exponential increase of ski resorts and skiing/snowboarding participation in such a short time, China has become the world’s largest junior ski market. However, compared with the ski resorts operations in the US, two critical areas that have not received due attention or investment are Ski/Snowboard Instructors and Ski Patrol programs.

Ski safety programs have been essentially neglected during this period of rapid development [98]. Safety is critically important for any sport or recreational activity. Ski and snowboarding are winter activities that are potentially beneficial to health and fitness; however, they present with a significant risk of injury. If not well managed, high injury rates will impact the ski industry and its development negatively. Founded in 1938, the National Ski Patrol (NSP) is the largest winter education organisation in the world and plays a critical role in the ski resorts in the US. The NSP provides education, outreach, and credentialing related to outdoor recreation and safety. The NSP has assisted to establish patrol organisations in Canada, Korea, New Zealand, Israel, Argentina, etc. To ensure sustainable development of snow sport and better prepare for hosting a successful 2022 Olympic and Paralympic Winter Games, a quality ski patrol and rescue system is in urgent need in China.

## Conclusion

The 2022 Winter Olympics in Beijing provides China opportunities to develop winter sport and research in sport science. Success in endurance sport like cross-country skiing and long-track skating requires considerable training volume and attention to skill and technique and mental skills, as well as novel equipment and better nutritional strategies. Endurance training in cross-country skiing includes much training at low ambient temperatures. Although it is well established that hypothermia impairs endurance performance in winter sport, the underlying physiological mechanisms are not fully characterised or understood. Strategies to prevent asthma and nutritional requirements and recommendations established for warm weather athletes are also, in general, likely to be applied to winter sport athletes. Further attention and research are required around the effects of cold, low sunlight exposure, protein requirements and recovery nutrition to optimise endurance performance in winter sport.
